# Effect of hyperbaric oxygen on the growth and susceptibility of facultatively anaerobic bacteria and bacteria with oxidative metabolism to selected antibiotics

**DOI:** 10.1007/s12223-023-01120-5

**Published:** 2023-12-15

**Authors:** Dittmar Chmelař, Miroslav Rozložník, Michal Hájek, Nikol Pospíšilová, Jozef Kuzma

**Affiliations:** 1 Institute of Laboratory Medicine, Institute of Microbiology, Faculty of Medicine, University of Ostrava, Ostrava, Czech Republic; 2 Centre for Hyperbaric Medicine of Faculty of Medicine and Ostrava City Hospital, Faculty of Medicine, University of Ostrava, Ostrava, Czech Republic; 3 Centre of Hyperbaric Medicine, Ostrava City Hospital, Nemocnicni 20, Ostrava, 728 80 Czech Republic

**Keywords:** Antibiotic susceptibility testing, Hyperbaric oxygenation, Facultatively anaerobic bacteria, Pseudomonads

## Abstract

Wild strains of *Pseudomonas aeruginosa*, *Escherichia coli*, *Klebsiella pneumoniae*, and *Proteus mirabilis* were tested in an experimental hyperbaric chamber to determine the possible effect of hyperbaric oxygen on the susceptibility of these strains to the antibiotics ampicillin, ampicillin + sulbactam, cefazolin, cefuroxime, cefoxitin, gentamicin, sulfamethoxazole + trimethoprim, colistin, oxolinic acid, ofloxacin, tetracycline, and aztreonam during their cultivation at 23 °C and 36.5 °C. Ninety-six-well inoculated microplates with tested antibiotics in Mueller–Hinton broth were cultured under standard incubator conditions (normobaric normoxia) for 24 h or in an experimental hyperbaric chamber (HAUX, Germany) for 24 h at 2.8 ATA of 100% oxygen (hyperbaric hyperoxia). The hyperbaric chamber was pressurised with pure oxygen (100%). Both cultures (normoxic and hyperoxic) were carried out at 23 °C and 36.5 °C to study the possible effect of the cultivation temperature. No significant differences were observed between 23 and 36.5 °C cultivation with or without the 2-h lag phase in *Pseudomonas aeruginosa*, *Escherichia coli*, *Klebsiella pneumoniae*, and *Proteus mirabilis*. Cultivation in a hyperbaric chamber at 23 °C and 36.5 °C with or without a 2-h lag phase did not produce significant changes in the minimum inhibitory concentration (MIC) of *Escherichia coli*, *Klebsiella pneumoniae*, and *Proteus mirabilis*. For the tested strains of *Pseudomonas aeruginosa*, the possible effect of hyperbaric oxygen on their antibiotic sensitivity could not be detected because the growth of these bacteria was completely inhibited by 100% hyperbaric oxygen at 2.8 ATA under all hyperbaric conditions tested at 23 °C and 36.5 °C. Subsequent tests with wild strains of pseudomonads, burkholderias, and stenotrophomonads not only confirmed the fact that these bacteria stop growing under hyperbaric conditions at a pressure of 2.8 ATA of 100% oxygen but also indicated that inhibition of growth of these bacteria under hyperbaric conditions is reversible.

## Introduction

Currently, there is a growing need for new antibiotics for highly resistant microbial infectious agents. In addition to the search for new antimicrobials, new combinations of antibiotics are being proposed to treat multidrug-resistant bacterial infections.

Pure oxygen breathing under elevated pressure, called hyperbaric oxygen therapy (HBOT), has become the standard treatment for the treatment of infected wounds, especially for deep and chronic infections such as necrotising fasciitis, osteomyelitis, chronic soft tissue infections, and diabetic foot infections (Hájek et al. 2017). As a result of the hyperoxic conditions induced by HBOT, several physiological and biochemical modulations occur that stimulate antimicrobial effects. HBOT has been shown to have bactericidal and bacteriostatic effects on aerobic and primarily anaerobic bacteria, to enhance the antimicrobial effects of the immune system, and to have additive or synergistic effects with some antimicrobial agents. Since antimicrobial drugs tend to lose their effect over time as a result of resistance development, HBOT may represent a very effective antimicrobial therapy in which resistance cannot be easily achieved. Despite scientific and clinical evidence of the synergistic effect of HBOT and antibiotics, little is known about the underlying molecular mechanisms. The aim of this study was to investigate the effect of hyperbaric oxygen on pathogenic *Escherichia coli* (ESCO), *Pseudomonas aeruginosa* (PSAE), *Proteus mirabilis* (PRMI), and *Klebsiella pneumoniae* (KLPN) in combination with selected antibiotics. To this end, the effect of hyperbaric oxygen alone and the effect of the combined effect of HBOT and selected antibiotics were evaluated.

The most serious infections caused by Gram-negative bacteria occur in healthcare facilities and are most commonly caused by bacteria of the Enterobacteriaceae family (mostly *Klebsiella pneumoniae*), *Pseudomonas aeruginosa*, and bacteria of the genus *Acinetobacter*. The discovery and development of alternative therapeutic strategies that represent novel approaches against multidrug-resistant bacteria are gaining increasing attention. In addition to antibiotics (Martelli and Giacomini 2018), alternative potential agents include antimicrobial peptides (Chung and Khanum 2017), metal–metal nanoparticles (Hemeg 2017), *quorum sensing* inhibitors (Brackman and Coeney 2015), and hybrid antibiotics (Domalon et al. 2018). Hyperbaric oxygen therapy (HBOT) is a therapy based on inhalation of pure medical oxygen under elevated ambient pressure. HBOT is commonly administered as a primary or alternative therapy for various diseases such as infections.

HBOT has a direct elimination effect on anaerobic bacteria, enhances the antimicrobial effects of the immune system, and has an additive or synergistic effect with some antimicrobial agents. HBOT has been described as a useful procedure for a variety of infections, especially deep and chronic infections such as necrotising and chronic soft tissue infections (Memar et al. 2019). Hyperoxia (98% O_2_ at 2.8 absolute ATA—approximately 284.6 kPa) has been shown to enhance the effect of nitrofurantoin, sulfamethoxazole, trimethoprim, gentamicin, and tobramycin in *Escherichia coli* strains (serotype 018 and ATCC 25922). Similarly, the ability of hyperoxia to enhance the efficacy of aminoglycosides on *Pseudomonas aeruginosa* ATCC 27853 was tested. The MIC values of the tested bacterial strains showed that hyperoxia did not affect the efficacy of gentamicin and tobramycin, but increased the antimicrobial activity of nitrofurantoin and trimethoprim and synergistically enhanced the bacteriostatic effect of tobramycin. Hyperbaric oxygen also enhanced the bacteriostatic effects of tobramycin on *Pseudomonas aeruginosa* (Muhwich et al. 1989). Recently, HBOT has also been reported to reduce *Pseudomonas aeruginosa* viability in a way dependent on oxygen pressure (Rozložník et al. 2020).

Bacteria exhibit characteristic markers of apoptosis (including phosphatidylserine exposure and DNA fragmentation) associated with stress that triggers cell death, specifically in the form of bactericidal antibiotic treatment. Prokaryotic organisms have mechanisms to disassemble and mark dying cells in response to various noxious stimuli in the form of elaborate multilevel proteolytic regulation of these functions (Dwyer et al. 2012).

## Material and methods

### Principal methods

#### Clinical bacterial isolates

To evaluate the effect of hyperbaric hyperoxia on antibiotic sensitivity, four pathogenic bacteria isolated from hospital patients (wild types) from the operational area of the Agel Laboratory of Vitkovice Hospital in Ostrava were used, namely *Pseudomonas aeruginosa*, *Escherichia coli*, *Klebsiella pneumoniae*, and *Proteus mirabilis*. The initial microbial evaluation showed good antibiotic sensitivity in *Escherichia coli* and *Proteus mirabilis*. *Klebsiella pneumoniae* was producing widespread beta-lactamases of ESBL type (extended-spectrum beta-lactamases). The tested strain of *Pseudomonas aeruginosa* was a wild strain that did not produce beta-lactamase. All bacterial strains tested were identified using the MALDI-TOF MS system (Bruker Daltonics, Germany) at a probability level of 99%.

#### Antibiotic susceptibility testing

Antibiotic susceptibility testing was assessed using the broth dilution method in microplates. Ninety-six-well plates (G-1 type for Gram-negative bacteria) were obtained from Trios (Czech Republic) and stored at − 60 °C until unfrozen at room temperature and used. G-1 microplates are standardly used to evaluate the minimum inhibitory concentration (MIC) in bacterial strains in Czech reference laboratories. The composition and concentration of antibiotics are shown in Table 1.

**Table 1:** Ninety-six-well microplate G-1 setup. Numbers represent concentration of a given antibiotic (mg/L). *AMP* ampicillin, *AMS* ampicillin + sulbactam, *CZL* cefazolin, *CRX* cefuroxime, *CXT* cefoxitin, *GEN* gentamicin, *COT* sulfamethoxazole + trimethoprim, *COL* colistin, *OXO* oxolinic acid, *OFL* ofloxacin, *TET* tetracycline, *AZT* aztreonam, *GC* growth control

AMP	AMS	CZL	CRX	CXT	GEN	COT	COL	OXO	OFL	TET	AZT
64	64	64	64	64	32	256	32	128	8	64	GC
32	32	32	32	32	16	128	16	64	4	32	16
16	16	16	16	16	8	64	8	32	2	16	8
8	8	8	8	8	4	32	4	16	1	8	4
4	4	4	4	4	2	16	2	8	0.5	4	2
2	2	2	2	2	1	8	1	4	0.25	2	1
1	1	1	1	1	0.5	4	0.5	2	0.125	1	0.5
0.5	0.5	0.5	0.5	0.5	0.25	2	0.25	1	0.063	0.5	0.25

### The procedure

All bacterial strains were grown on Mueller–Hinton agar (Trios, Czech Republic) for 24 h prior to each experiment. Subsequently, the inoculum was prepared by dilution of bacterial colonies in physiological solution at a concentration of 1.5 × 10^8^ CFU/mL (grade 1 of the McFarland standard). The turbidity was checked by densimeter (Erba-Lachema, Czech Republic). After pouring the inoculum into sterile Petri dishes, Mueller–Hinton broth 96-well microplates with antibiotics (Trios, Czech Republic) were inoculated with a sterile inoculator. After inoculation, the 96-well microplates were exposed to normobaric and hyperbaric conditions for *Escherichia coli strains*, *Klebsiella pneumoniae*, *Proteus mirabilis*, and *Pseudomonas aeruginosa*. All experiments were carried out repeatedly (three times) for every bacterial strain and every experimental setup.

### Methods of cultivation

The inoculated 96-well G-1 microplates were grown in standard incubator conditions (normobaric normoxia) conditions for 24 h or in an experimental hyperbaric chamber (HAUX, Germany) for 24 h at 2.8 ATA of 100% oxygen (hyperbaric hyperoxia).

Various pressure units are mentioned in the literature, although international agreements have been made on a standardised nomenclature for many years. In the literature on hyperbaric or diving medicine, non-SI units such as ATA, atm, bar, metres of water, and feet of water (fsw) are commonly used. Similarly, units of millimetres of Hg are used in many countries for blood pressure and blood gases. The ATA (atmospheres absolute) unit is the most commonly used in hyperbaric medicine, as it expresses the sum of atmospheric pressure and gauge pressure within pressure systems including the hyperbaric chamber (or hydrostatic pressure while diving or working underwater).

The hyperbaric chamber was pressurised with pure oxygen (100% medical grade). Both cultures (normoxic and hyperoxic) were carried out at 36.5 °C or 23 °C to study the possible effect of the cultivation temperature. Furthermore, to evaluate the effect of the 2-h lag phase, the microplates were stored at room temperature (23 °C) or 36.5 °C (normobaric and normoxic) before growing in an incubator or hyperbaric chamber. The 2-h lag phase was not estimated but was determined equally for all strains tested. It was chosen deliberately taking into account both the usual lag time of the tested bacteria and, above all, the possible influence of increased hyperbaric oxygen pressure during the lag phase on the viability of the tested bacteria.

### Transfer of methods

As *Pseudomonas aeruginosa* was found to stop growing at 2.8 ATA, further cultures were carried out with this bacterium at pressures of 1. 9 and 2.4 ATA to determine the pressure limit at which these bacteria stop growing.

## Results

**Fig. 1:**
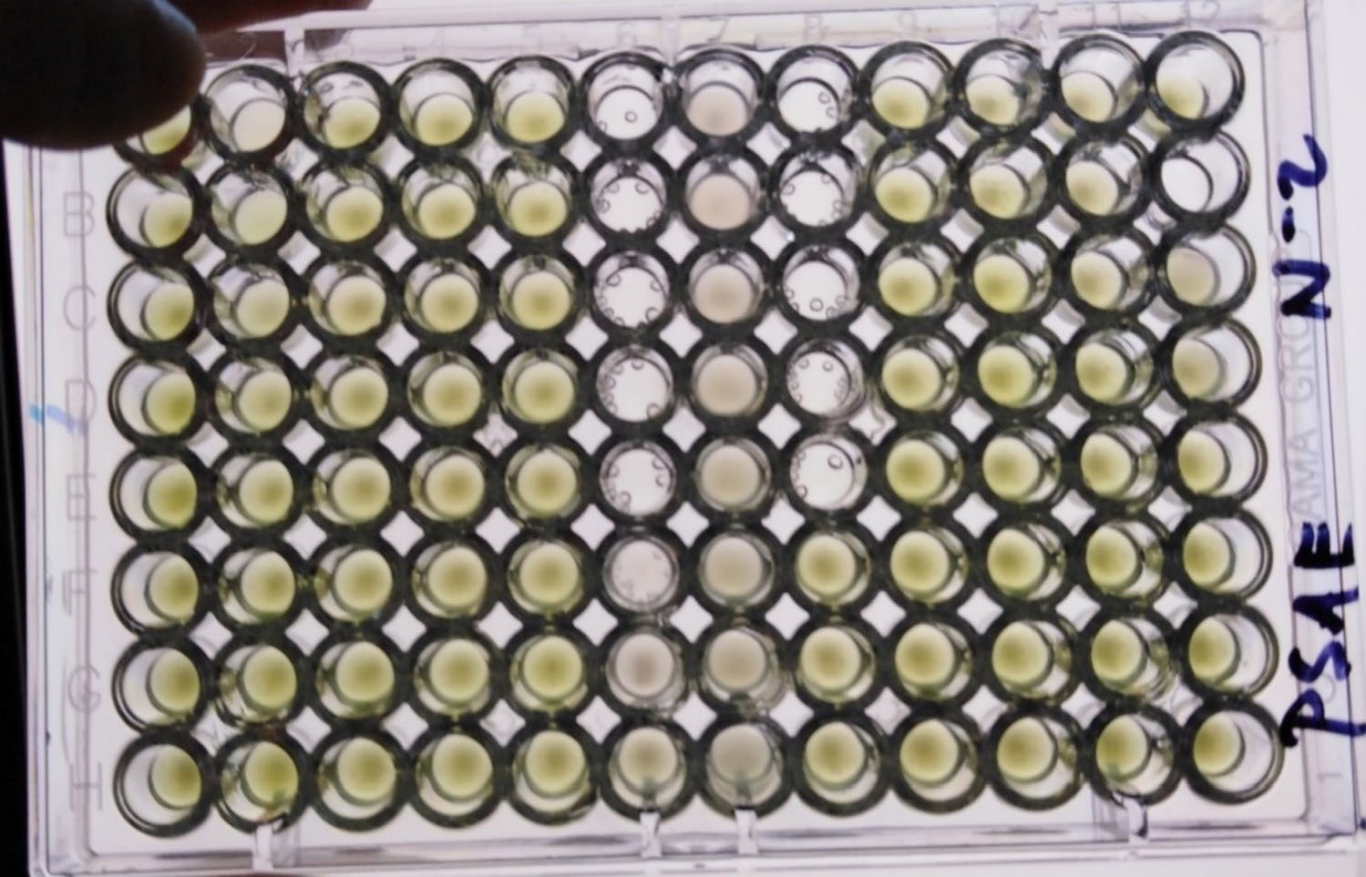
Minimum inhibitory concentration (MIC) of the tested antibiotics in the 96-well microplate set-*Pseudomonas aeruginosa*-culture under normobaric conditions (pressure 1 ATA, 21% oxygen)

**Fig. 2:**
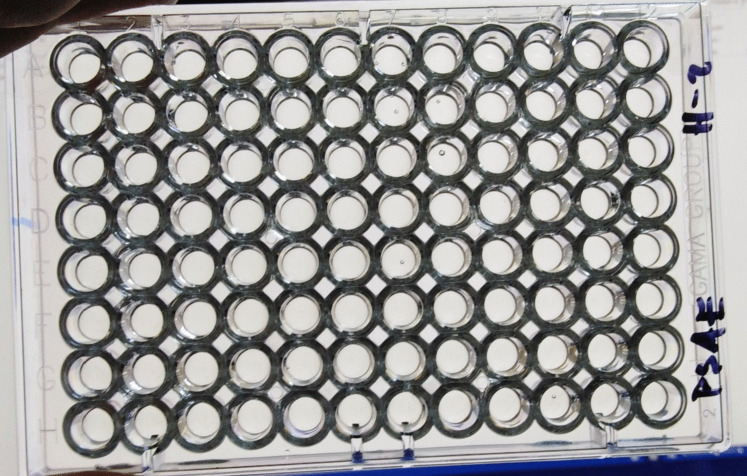
Minimum inhibitory concentration (MIC) of the tested antibiotics in the 96-well microplate set-*Pseudomonas aeruginosa*-culture under hyperbaric conditions (pressure 2.8 ATA, 100% oxygen)—no growth was observed, even in the control well without antibiotic (well A12)

The results of the determination of susceptibility to selected antibiotics under normobaric and hyperbaric conditions for *Escherichia coli strains*, *Klebsiella pneumoniae*, *Proteus mirabilis*, and *Pseudomonas aeruginosa* are presented in Tables 2, 3, 4, and 5 and Figs. 1 and 2. No significant differences were observed between the cultivation at 23 and 36.5 °C with or without 2-h lag phase under normobaric conditions in *Pseudomonas aeruginosa*, *Escherichia coli*, *Klebsiella pneumoniae*, and *Proteus mirabilis*. The cultivation in the hyperbaric chamber at 23 and 36.5 °C with or without 2-h lag phase did not induce significant changes in minimal inhibitory concentration (MIC) in *Escherichia coli*, *Klebsiella pneumoniae*, and *Proteus mirabilis*. The susceptibility profiles obtained to the antibiotics tested indicate that hyperbaric hyperoxia (pressure 2.8 ATA, 100% oxygen) has a significant effect on the growth of *Pseudomonas aeruginosa*. The growth of these bacteria was inhibited by 100% hyperbaric oxygen under all hyperbaric conditions tested at 23 °C and at 36.5 °C, including cultivation under hyperbaric conditions with an initial 2-h lag phase of cultivation under normobaric conditions. For the other bacteria tested, no differences in sensitivity to antibiotics tested were found under normobaric and hyperbaric conditions. Also, different lag phase times and cultivation at different temperatures did not show differences in sensitivity to antibiotics tested. The observed changes in the sensitivity profile are only minimal (for *Klebsiella pneumoniae* for the antibiotics COT (cotrimoxazole) and OXO (oxolinic acid); for *Proteus mirabilis* for the antibiotics COL (colistin) and TET (tetracycline)) and must be attributed to random fluctuations in the actual determination of sensitivity, especially when the changes are within the range of a single well (one dilution of the antibiotic).

**Table 2:** Minimum inhibitory concentration (MIC, mg/L)—*Escherichia coli* (ESCO) after cultivation under normobaric and hyperbaric conditions

	23 N	23 H	36.5 N	36.5 H	36.5 b. lag
AMP	64	64	64	64	64
AMS	8	8	8	4	2
CZL	8	8	8	4	8
CRX	4	4	4	2	2
CXT	4	4	4	2	2
GEN	4	4	4	1	1
COT	4	4	4	4	8
COL	0.5	0.5	0.5	0.5	0.5
OXO	0.5	1	0,5	0.5	0.5
OFL	0.125	0.125	0.125	0,5	0.125
TET	4	2	4	4	0.5
AZT	0.25	0.25	0.25	0.25	0.25

Explanations: 23 N, cultivation at 23 °C, normobaric conditions; 23 H, cultivation at 23 °C, hyperbaroxia (2.8 ATA, 100% medical oxygen); 36.5 N, cultivation at 36.5 °C, normobaric conditions; 36.5 H, cultivation at 36.5 °C, hyperbaroxia with 2-h lag phase, hyperbaroxia (2.8 ATA, 100% medical oxygen); 36.5 b. lag, cultivation at 36.5 °C, hyperbaroxia without 2-h lag phase, hyperbaroxia (2.8 ATA, 100% medical oxygen)

**Table 3:** Minimum inhibitory concentration (MIC, mg/L)—*Klebsiella pneumoniae* (KLPN) after cultivation under normobaric and hyperbaric conditions

	23 N	23 H	36.5 N	36.5 H	36.5 b. lag
AMP	64	64	64	64	64
AMS	4	4	8	8	8
CZL	64	64	64	64	64
CRX	64	64	64	64	64
CXT	2	4	8	2	2
GEN	32	32	32	32	32
COT	2	2	32	16	8
COL	0,5	2	2	0.25	4
OXO	32	32	64	64	32
OFL	0.125	0.125	1	2	1
TET	16	8	8	8	8
AZT	16	16	16	16	16

Explanations: 23 N, cultivation at 23 °C, normobaric conditions; 23 H, cultivation at 23 °C, hyperbaroxia (2.8 ATA, 100% medical oxygen); 36.5 N, cultivation at 36.5 °C, normobaric conditions; 36.5 H, cultivation at 36.5 °C, hyperbaroxia with 2-h lag phase, hyperbaroxia (2.8 ATA, 100% medical oxygen); 36.5 b. lag, cultivation at 36.5 °C, hyperbaroxia without 2-h lag phase, hyperbaroxia (2.8 ATA, 100% medical oxygen)

**Table 4:** Minimum inhibitory concentration (MIC, mg/L)—*Proteus mirabilis* (PRMI) after cultivation under normobaric and hyperbaric conditions

	23 N	23 H	36.5 N	36.5 H	36.5 b. lag
AMP	2	4	1	1	1
AMS	2	2	1	1	2
CZL	8	8	8	8	8
CRX	2	2	1	1	2
CXT	8	8	4	4	4
GEN	2	1	1	1	1
COT	4	4	4	2	4
COL	64	64	32	32	64
OXO	2	1	1	1	1
OFL	0.125	0.125	0.063	0.063	0.125
TET	64	32	64	64	32
AZT	0.5	0.25	0.25	0.25	0.25

Explanations: 23 N, cultivation at 23 °C, normobaric conditions; 23 H, cultivation at 23 °C, hyperbaroxia (2.8 ATA, 100% medical oxygen); 36.5 N, cultivation at 36.5 °C, normobaric conditions; 36.5 H, cultivation at 36.5 °C, hyperbaroxia with 2-h lag phase, hyperbaroxia (2.8 ATA, 100% medical oxygen); 36.5 b. lag, cultivation at 36.5 °C, hyperbaroxia without 2-h lag phase, hyperbaroxia (2.8 ATA, 100% medical oxygen)

**Table 5:** Minimum inhibitory concentration (MIC, mg/L)—*Pseudomonas aeruginosa* (PSAE) after cultivation under normobaric and hyperbaric conditions

	23 N	23 H	36.5 N	36.5 H	36.5 b. lag
AMP	64	No growth	64	No growth	No growth
AMS	64	No growth	64	No growth	No growth
CZL	64	No growth	64	No growth	No growth
CRX	64	No growth	64	No growth	No growth
CXT	64	No growth	64	No growth	No growth
GEN	2	No growth	2	No growth	No growth
COT	64	No growth	64	No growth	No growth
COL	0.5	No growth	0.5	No growth	No growth
OXO	64	No growth	64	No growth	No growth
OFL	16	No growth	16	No growth	No growth
TET	16	No growth	16	No growth	No growth
AZT	16	No growth	16	No growth	No growth

Explanations: 23 N, cultivation at 23 °C, normobaric conditions; 23 H, cultivation at 23 °C, hyperbaroxia (2.8 ATA, 100% medical oxygen); 36.5 N, cultivation at 36.5 °C, normobaric conditions; 36.5 H, cultivation at 36.5 °C, hyperbaroxia with 2-h lag phase, hyperbaroxia (2.8 ATA, 100% medical oxygen); 36.5 b. lag, cultivation at 36.5 °C, hyperbaroxia without 2-h lag phase, hyperbaroxia (2.8 ATA, 100% medical oxygen)

Taking into account the surprising results of no growth of the tested strain of *Pseudomonas aeruginosa* under hyperbaric conditions at 2.8 ATA, the hyperbaric pressure value of 100% oxygen was determined at which the growth of *Pseudomonas aeruginosa* stops. For this purpose, 2 wild strains of *Pseudomonas aeruginosa* as well as 2 wild strains of *Burkholderia cepacia* and 2 wild strains of *Stenotrophomonas maltophilia* were selected. All strains came from the Agel Laboratory of Vitkovice Hospital in Ostrava. These selected strains were identified using the MALDI-TOF MS identification system (Bruker Daltonics, Germany) at the 99% identification level. The tested bacterial strains were cultured for 24 h on Mueller–Hinton agar (HiMedia, India). Subsequently, they were inoculated in fresh Mueller–Hinton medium and cultured under normobaric conditions at 36.5 °C and hyperbaric conditions at different pressures of 100% oxygen for 24 h in the hyperbaric chamber. The results of the bacteria grown show that the tested strains of bacteria with oxidative metabolism grow at pressures of 1.9 ATA and 2.4 ATA at 100% oxygen and stop growing at 2.8 ATA. The tested strains of pseudomonads, burkholderias, and stenotrophomonads were also grown in the HAUX experimental hyperbaric chamber at a normobaric pressure of 1.0 ATA at a 100% medical oxygen concentration. All tested bacterial strains showed growth after 24 h of cultivation, indicating that the cause of the growth arrest of the tested bacteria is not the 100% oxygen concentration itself.

All tested bacterial strains on Petri plates with Mueller–Hinton agar, which were grown under hyperbaric conditions in the range of 1.9–2.8 ATA of 100% oxygen, were cultured in a thermostat under normobaric conditions at 36.5 °C for 24 h immediately after removal from the hyperbaric chamber. All of these bacteria showed the same growth after this time as after cultivation under the same normobaric conditions. Thus, stopping growth of tested pseudomonads, burkholderias, and stenotrophomonads is clearly reversible. Detection of growth or non-growth of each tested bacterial species was detected visually by looking at Petri dishes with inoculated bacterial strains in individual lines by the cross-streaking plate technique. The growth for 24 h of all wild strains of bacteria with oxidative metabolism (PSAE (*Pseudomonas aeruginosa*); BUCE (*Burkholderia cepacia*); STMA (*Stenotrophomonas maltophilia*)) under different pressure conditions was positive up to hyperbaric hyperoxia conditions of 2.4 ATA. No growth was observed under the same conditions but at a pressure of 2.8 ATA.

## Discussion

### Effect of hyperbaric oxygen on bacterial growth and susceptibility to antibiotics

The results of this work indicate that bacteria with oxidative metabolism, that is, pseudomonads, burkholderias, and stenotrophomonads, stop growing under hyperbaric conditions at a pressure of 2.8 ATA in an environment of pure oxygen. The effect of hyperbaric hyperoxia is reversible as these bacterial strains grow again under normobaric conditions.

Some antibiotics cause oxidative stress to bacteria. After the application of antibiotics (beta-lactam or fluoroquinolone antibiotics), increased expression of genes, regulators of oxidative stress, has been detected in bacteria. The action of these antibiotics slows respiration, leading to an acceleration of side reactions associated with the production of reactive oxygen species (ROS). The slowing of metabolism leads to the accumulation of ADP, as it is not used. The change in the ATP/ADP ratio is an indicator of an imbalance between energy production and consumption (Akhova and Tkachenko 2014). Other studies confirm the bactericidal effect of antibiotics even under anaerobic conditions. This points to the fact that ROS generation is not the sole cause of antibiotic-induced bacterial death, but is an interplay of multiple mechanisms (Keren et al. 2013).

Standard antimicrobial susceptibility tests are performed in vitro under normal oxygen conditions in the room to predict the in vivo effectiveness of antimicrobial therapy. The purpose of one recent study was to perform a comprehensive analysis of the effect of different oxygen levels on the susceptibility to antibiotics of two strains of *Staphylococcus aureus*, *Pseudomonas aeruginosa*, and *Klebsiella pneumoniae*. Anoxic conditions were found to cause a reduced sensitivity of bacteria to aminoglycoside antibiotics in four of the six bacteria used in the study. Additionally, oxygen limitation decreased the susceptibility of *Pseudomonas aeruginosa* strains and *Klebsiella pneumoniae* strains to piperacillin/tazobactam and azithromycin, respectively. In contrast, five of the six bacteria became more susceptible to tetracycline antibiotics under oxygen-limiting conditions. It was shown that an enriched oxygen environment might favour the growth and biofilm formation of some pathogens, but this does not necessarily reflect a significant difference in antimicrobial efficacy. These data show the importance of considering the potential in vivo oxygen levels within the infection site when setting susceptibility breakpoints to evaluate the therapeutic potential of a drug and its effect on the antibiotic sensitivity of the pathogen. Authors conclude that the use of clinically relevant oxygen environments should be a parameter in antimicrobial susceptibility testing and breakpoints should be established accordingly. This would help physicians make better therapeutic decisions by more accurately predicting the susceptibility of pathogens in vivo, leading to better clinical outcomes (Gupta et al. 2016).

### Clinical effects of hyperbaric oxygen

Based on the pathogenesis and pathophysiological conditions of infectious endocarditis (IE), there are some important mechanisms and effects of HBOT in relation to infection and inflammation in general. There are some aspects and impact of HBOT in relation to the host response, tissue hypoxia, biofilms, antibiotics, and pathogens. New therapeutic options in IE are much needed, and adjunctive HBOT could be a therapeutic option in certain patients to decrease morbidity and mortality and improve the long-term outcome of this severe disease. Two animal studies have shown a beneficial effect of HBOT in IE, but so far, no clinical study has evaluated the feasibility of HBOT in IE. Intermittent HBOT demonstrates multiple beneficial effects, dampening the detrimental host–pathogen interactions in IE. HBOT remains one of the most effective clinical means of oxygenation delivery to deep infections of vital tissues. Data using the present guidelines for the antibiotic treatment of IE indicate the need for improved treatment strategies. HBOT is a promising candidate as an adjunctive treatment strategy due to the multifaceted effects and the pathophysiology of IE (Lerche et al. 2022).

When the effect of hyperbaroxia is evaluated, it is assumed that, in view of the known facts about the neutralisation of gases by bacteria, the positive effect in the treatment of mixed infections is not achieved by the primary effect of hyperbaric oxygen on pathogenic bacteria that are the provokers of these diseases but by its secondary effect consisting mainly in a significant stimulation of the patient’s immune system.

### Effect of hyperbaric oxygen on obligate aerobes and anaerobes

Only a few genera of bacteria (*Rhizobium*, *Azomonas*, *Beijerinckia*, and *Azotobacter*—the so-called tuber bacteria) are able to use nitrogen gas during its fixation in the root system of mainly leguminous plants, and they are able to reduce the triple bond in the atmospheric nitrogen molecule. It is then converted to an inorganic compound (ammonia). Oxygen increases the ability of bacteria to eliminate the effect of some antibiotics, for example, tobramycin, vancomycin, and sulfonamides. Some aminoglycosides use an oxygen-dependent transport system to enter bacterial cells. The increased oxygen pressure improves this transmembrane transport of antibiotics into bacterial cells. Apparently, the enormously increased pressure of 100% pure oxygen results in such a pronounced production of oxygen radicals (oxidants), substances with strong oxidative and cidal effects that in bacteria with oxidative metabolism, such as strains of *Pseudomonas aeruginosa*, the amount of hyperbaric oxygen has the effect of stop by their growth. These bacteria do not possess an alternative form of metabolism that would allow them to persist under such altered conditions. There is no production of superoxide dismutases, peroxidases, or catalases to inhibit oxygen radicals. Their catalytic systems are not powerful enough to cope with the supercritical amounts of oxygen radicals produced. Facultatively, anaerobic bacteria in environments where there are large amounts of substances with strong oxidative effects are likely to switch their metabolic pathways to anaerobic respiration, allowing them to survive in these environments with large amounts of oxygen radicals (Lu and Imlay 2021).

Another possible explanation for the fact that strains of pseudomonads, burkholderias, and stenotrophomonads do not grow under hyperbaric conditions in an atmosphere of 100% oxygen and a pressure of 2.8 ATA may be that the plasmids of these strains carrying genes involved in the knockout of genes responsible for growth are pushed out of the cells by the high pressure and thus cannot be expressed. In view of the very surprising results obtained, more detailed genomic investigations of *Pseudomonas aeruginosa* genes will be necessary to verify their expression or elimination of these genes, which may be the cause of elimination of growth of these bacterial cells under hyperbaric conditions in the presence of 100% oxygen.

## Conclusions

The results of this work indicate that bacteria with oxidative metabolism, i.e. pseudomonads, burkholderias, and stenotrophomonads, stop to grow under hyperbaric conditions at a pressure of 2.8 ATA in an environment of 100% oxygen. The effect of hyperbaric hyperoxia is reversible as these bacterial strains grow again under normobaric conditions.

## Data Availability

The data that support the findings of this study are available from the corresponding author upon reasonable request.

## Abbreviations

ADP: Adenosine diphosphate
ATP: Adenosine triphosphate
ATA: Atmospheres absolute
CFU: Colony-forming unit
DNA: Deoxyribonucleic acid
HBOT: Hyperbaric oxygen therapy
BUCE: *Burkholderia cepacia*
ESCO: *Escherichia coli*
KLPM: *Klebsiella pneumoniae*
PSAE: *Pseudomonas aeruginosa*
PRMI: *Proteus mirabilis*
STMA: *Stenotrophomonas maltophilia*
IE: Infective endocarditis
MALDI-TOF MS: Matrix-assisted laser desorption ionization–time-of-flight mass spectrometry
MALDI: Ionization technique
TOF: Time of flight, type of ion detection
MS: Mass spectrometry
MIC: Minimum inhibitory concentration
ROS: Reactive oxygen species
